# PEZy-miner: An artificial intelligence driven approach for the discovery of plastic-degrading enzyme candidates

**DOI:** 10.1016/j.mec.2024.e00248

**Published:** 2024-09-05

**Authors:** Renjing Jiang, Zhenrui Yue, Lanyu Shang, Dong Wang, Na Wei

**Affiliations:** a Department of Civil and Environmental Engineering, University of Illinois Urbana-Champaign, Urbana, IL, 61801, United States; b School of Information Sciences, University of Illinois Urbana-Champaign, Champaign, IL, 61820, United States

**Keywords:** Machine learning, Plastic degradation, Enzyme discovery, Protein language model, Confidence and uncertainty estimation

## Abstract

Plastic waste has caused a global environmental crisis. Biocatalytic depolymerization mediated by enzymes has emerged as an efficient and sustainable alternative for plastic treatment and recycling. However, it is challenging and time-consuming to discover novel plastic-degrading enzymes using conventional cultivation-based or omics methods. There is a growing interest in developing effective computational methods to identify new enzymes with desirable plastic degradation functionalities by exploring the ever-increasing databases of protein sequences. In this study, we designed an innovative machine learning-based framework, named PEZy-Miner, to mine for enzymes with high potential in degrading plastics of interest. Two datasets integrating information from experimentally verified enzymes and homologs with unknown plastic-degrading activity were created respectively, covering eleven types of plastic substrates. Protein language models and binary classification models were developed to predict enzymatic degradation of plastics along with confidence and uncertainty estimation. PEZy-Miner exhibited high prediction accuracy and stability when validated on experimentally verified enzymes. Furthermore, by masking the experimentally verified enzymes and blending them into homolog dataset, PEZy-Miner effectively concentrated the experimentally verified entries by 14∼30 times while shortlisting promising plastic-degrading enzyme candidates. We applied PEZy-Miner to 0.1 million putative sequences, out of which 27 new sequences were identified with high confidence. This study provided a new computational tool for mining and recommending promising new plastic-degrading enzymes.

## Highlights

• Machine learning is used to learn protein language and predict plastic degradation.
• Confidence and uncertainty estimation identifies top-ranked enzyme candidates.
• 27 out of 0.1 million sequences are discovered as promising candidates.
• PEZy-Miner is developed to help end-user mine for top enzymes candidates.

## 1 Introduction

The drastically increasing amount of plastic waste is causing an environmental crisis (Chang et al., 2020; Rochman et al., 2013; Smith et al., 2018). There is an urgent need of effective and innovative approaches for treating and recycling of post-consumer plastics to achieve waste valorization while meeting environmental quality goals (Wei and Zimmermann, 2017a). The biodegradation of plastics has become a focus of research due to high efficiency of biological enzymes under mild reaction conditions (Zhu et al., 2022). Furthermore, some enzymes have been reported to depolymerize plastics such as poly(ethylene terephthalate) (PET), polycaprolactone (PCL), and poly(lactic acid) (PLA) into monomers, which can then be recovered for recycling and upcycling to achieve a circular economy (Lu et al., 2022; Oh et al., 2022; Sourkouni et al., 2023).

Significant research progress has been made in searching for novel enzymes capable of degrading plastics, but discovering plastic-degrading enzymes remains a challenging task (Roohi et al., 2017; Zhu et al., 2022). Conventional cultivation-based methods involving enrichment or isolation of plastic-degrading microorganisms are limited to enzymes from culturable organisms, which correspond to less than 1% of the total microorganisms (Kim et al., 2022; Zhu et al., 2022), reducing the likelihood of discovering novel plastic-degrading enzymes. Omics techniques such as metagenomics and proteomics involve intensive bioinformatic analysis and experimental screening (Hajighasemi et al., 2018; Kim et al., 2022; Sturmberger et al., 2016). The time-consuming and labor-intensive nature of these approaches poses a critical challenge in efficiently discovering novel plastic degrading enzymes and thus limits our capability to address the problem of recycling various plastics.

There are growing efforts in using homology-based computational approaches to identify new enzymes with desirable plastic degradation functionalities by exploring the ever-increasing databases of protein sequences (Buchholz et al., 2022; Danso et al., 2018; Viljakainen and Hug, 2021b; Zrimec et al., 2021). Homology search is a strategy to find sequences that share a common evolutionary ancestor (Pearson, 2013). In the efforts of searching for new plastic-degrading enzyme candidates, Danso et al. (2018) searched one hundred thirty-three metagenomes at global scale and identified three hundred homologs as potential PET hydrolases. Buchholz et al. (2022) found three thousand PET-active enzymes and two thousand polyurethane (PU)-active homologs with a wide coverage of microbial species. Zrimec et al. (2021) identified over thirty thousand enzyme homologs with the potential to degrade ten different plastic types. However, the homologs were found in such a large number that it is extremely challenging to experimentally characterize all enzyme candidates. For example, in Danso et al. (2018)'s study, only four homologs were selected for experimental validation due to their sequence similarity to known PET-degrading enzymes, while the remaining candidates are underexplored. Therefore, it is desirable that the promising enzyme candidates with high likelihood of plastic degradation activity are shortlisted from the large number of homology search results as promising candidates for feasible experimental studies. To this end, a commonly used approach in prior research efforts is to infer enzyme functionality based on sequence similarity (Pearson, 2013; Tian and Skolnick, 2003). However, relying solely on sequence similarity may result in incorrect predictions as sequence similarity does not consistently correlate with enzyme plastic-degrading functionality (Jiang et al., 2023; Pearson, 2013; Viljakainen and Hug, 2021a). For example, the PETase discovered from *Ideonella sakaiensis* 201-F6 shared only 51% sequence similarity with a previously known PET hydrolase from *Thermobifida fusca* (Yoshida et al., 2016), although above 60% sequence identity was suggested to ensure function similarity (Tian and Skolnick, 2003). Therefore, there is a critical need to develop an effective method which is less dependent on sequence similarity and taxonomy to accurately predict enzyme activities in plastic degradation. So, an innovative approach in alternative to sequence similarity is needed to effectively predict enzyme function in plastic degradation and identify potential novel enzyme candidates.

Recently, artificial intelligence (AI), in particular machine learning (ML), has received increasing attention in protein modeling. ML methods, which involve systematic computational analysis capable of capturing hidden patterns from a massive amount of data to make predictions or decisions (Domingos, 2012; Liu et al., 2022; Mitchell and Mitchell, 1997), have been applied to protein function prediction (Yu et al., 2023), mutation identification (Hie et al., 2024; Lu et al., 2022; Shin et al., 2021), and protein design problems (Ferruz and Höcker, 2022; Ferruz et al., 2022; Madani et al., 2023). Among the various methods in ML tasks, large protein language models (pLMs) open a new door to approach protein-related tasks (Ferruz et al., 2022). A pLM learns protein language by interpreting a protein sequence as a sentence and the amino acid residues as single words, akin to natural language processing (NLP), which is a computational methodology for automated analysis and representation of human languages (Chowdhary, 2020; Ruffolo and Madani, 2024). With the extraordinary advances in NLP, the emerging pLMs are reshaping the protein-related research field (Ferruz and Höcker, 2022) and have shown promise in various biotechnological applications, such as generating protein sequences with a predictable function across diverse protein families (Madani et al., 2023), sampling unexplored regions of protein space to facilitate *de novo* design (Ferruz et al., 2022), and capturing protein language grammar for predicting mutational effects (Rives et al., 2021). However, the implementation of ML, especially pLMs, remains underexplored in enzymatic degradation of plastics.

**Fig. 1:**
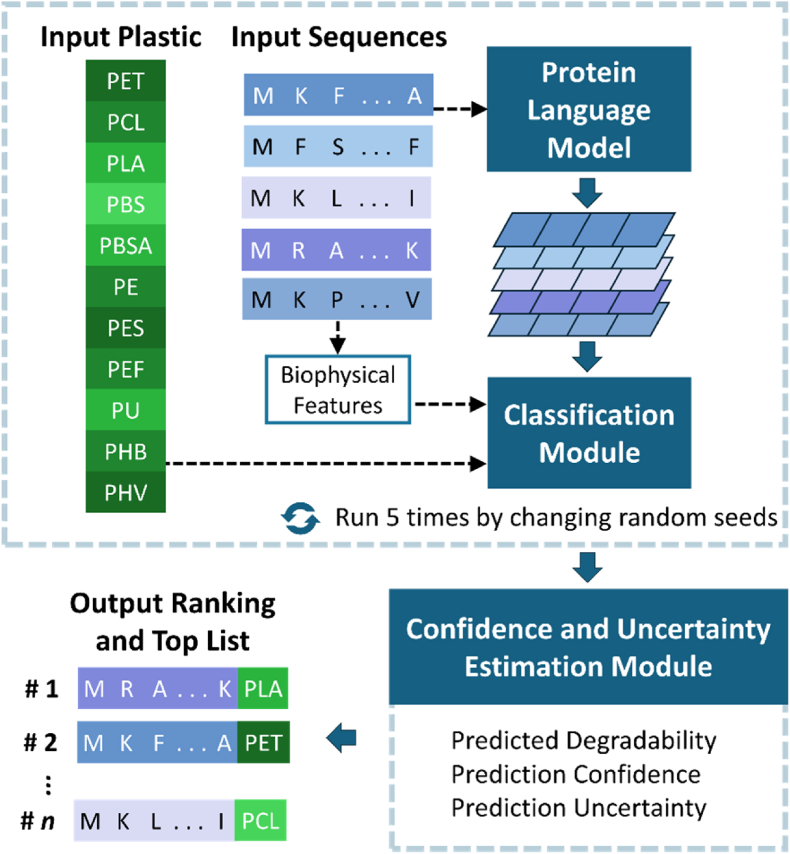
Overview of PEZy-Miner. The protein language model converted the input amino acid sequences into computer interpretable vectors. The classification module took as input the vectors, the biophysical features extracted from input sequences, and one-hot encoded plastic types for predicting the degradability of the input enzyme/plastic pairs. The confidence and uncertainty estimation module computed the confidence and uncertainty of the predictions. After five runs using different random seeds, the confidence and uncertainty estimation module integrated results to identify the top-ranked enzyme/plastic pairs common across the five runs.

In this study, we aimed to develop a reliable AI driven approach to discover promising enzyme candidates for plastic degradation by mining the database containing homologs to known plastic-degrading enzymes for a variety of common plastics. We designed a novel ML-based framework for mining enzymes with high potential in plastic degradation, named PEZy-Miner. The framework took amino acid sequences and plastic types of interest as inputs and return degradation ability predictions ranked by confidence and uncertainty (Fig. 1). Three key modules were integrated in PEZy-Miner: (1) a pLM to interpret and encode protein sequences, (2) a classification module to predict enzyme degradation of plastics, and (3) a confidence and uncertainty estimation module for ranking and generating the top list (Fig. 1). Three state-of-the-art pLMs and two classifiers were evaluated, and effectiveness of the confidence and uncertainty estimation was tested. The PEZy-Miner was then used to shortlist and rank promising plastic-degrading enzyme candidates from 0.3 million entries obtained from homology searches. The enzymes with experimentally verified ground-truth in plastic degradation capabilities were masked and blended into the homologous entries for evaluation and validation of the model prediction. This study demonstrated successful application of ML approaches to effectively infer enzyme functions in plastic degradation. The PEZy-Miner provides a new computational tool for mining and recommending promising enzyme candidates, which contributes to accelerating the discovery of new plastic-degrading enzymes.

## 2 Methods

### 2.1 Dataset preparation

The dataset about enzymatic degradation of plastics was created by manually collecting information from literature reports with experimental studies. Information included enzyme sequences, plastic types, and ground-truth labels (i.e., degradable or nondegradable) corresponding to an enzyme/plastic pair. It should be noted that degradation of certain plastics, such as PE, in literature reports sometimes only showed minimal observed degradation effects. Therefore, we only included the studies where the observed degradation effects were significant, such as over 10% weight loss (Bardají et al., 2019), over 10% CO_2_ production rate (defined as g-CO_2_ produced/g-CO_2_ when all the carbons of PE were mineralized into CO_2_) (Jeon and Kim, 2015, 2016), and conspicuous visual effect (Sanluis-Verdes et al., 2022). The dataset was referred to as “experimental dataset”. Our previous study developed an experimental dataset (Jiang et al., 2023), and the dataset was augmented in this study through literature review to incorporate the latest research on enzymatic plastic degradation. In all, the new experimental dataset in this study included 236 records of enzyme/plastic pairs with corresponding ground-truth labels, which were subsequently split into training dataset and testing dataset with a ratio of 7:3.

The “homologous dataset” was created by running Basic Local Alignment Search Tool (BLAST) searches at National Center for Biotechnology Information (NCBI) website (Altschul et al., 1990). To be specific, for every enzyme/plastic pair in the experimental dataset, the enzyme was used as a query sequence to search against the non-redundant protein sequences database. In every search, the max target sequences parameter was set to 5,000, and other algorithm parameters were by default (Supplementary Table S1). Search results were filtered by setting the threshold of percent identity to 30%, and the resulting sequences were assigned the same plastic type and label as the query sequences. In total, the homologous dataset including 277,702 enzyme/plastic pairs was constructed.

The “prescreened homologous dataset” was compiled by using the Contrastive Learning enabled Enzyme ANnotation (CLEAN) prediction tool (Yu et al., 2023). By analyzing the predicted Enzyme Commission (EC) numbers of the homologous dataset, we selected the homologs belonging to EC 1 oxidoreductase and EC 3 hydrolase with a confidence score larger than 0.99. To date, since plastic-degrading enzymes have consistently been characterized as EC 1 or EC 3 (Chen et al., 2020; Taniguchi et al., 2019; Temporiti et al., 2022; Viljakainen and Hug, 2021b), homologs of EC 1 and EC 3 were selected during prescreening. Additionally, the confidence estimation of CLEAN was informative and correlated with prediction accuracy (Yu et al., 2023). The cumulative accuracy of CLEAN was reported to be 0.5 when confidence score was set to (0, 0.9] and improved by 0.1 when confidence score was raised to (0, 1.0] (Yu et al., 2023). To ensure prediction accuracy of EC, the confidence cutoff of CLEAN was set to 0.99.

### 2.2 PEZy-miner development

#### 2.2.1 Encoding inputs via protein language models and one-hot method

In order to convert enzyme sequences into numerical vectors, pLMs were used. In analogy to natural language processing (NLP), which is a computational methodology for automated analysis and representation of human languages, a pLM learns protein language by interpreting a protein sequence as a sentence and the amino acids residues as single words (Chowdhary, 2020; Ruffolo and Madani, 2024). pLMs are utilized as encoder and/or decoder language models (Ruffolo and Madani, 2024). Encoders are used for learning numerical representations of protein sequences, which are subsequently incorporated into various downstream tasks, while decoders are employed for producing protein sequences (Ruffolo and Madani, 2024). In this study, encoders from three state-of-the-art pLMs were used, including ProtBERT (Elnaggar et al., 2022), ESM-2 (Lin et al., 2023), and RoBERTa (Liu et al., 2019). RoBERTa was originally designed for NLP, and it was pre-trained in this study using the homologous dataset, with configurations detailed in Supplementary Table S2. ProtBERT and ESM-2 were pre-trained by the developers using the UniRef100 and UniRef50 datasets respectively (Elnaggar et al., 2022; Lin et al., 2023), and therefore they were adopted without additional pre-training. After pre-training, representations of protein sequences were obtained. In particular, every pLM took as input a protein sequence *S* = [*A*_*1*_*, A*_*2*_*, …, A*_*N*_], where *A*_*i*_ was the amino acid at position *i* in the sequence. The input sequence was processed by the encoder through embedding layers, and the internal ‘summary’ of the sequence that had been learned by the encoder was obtained as a 30-length vector and used as the ‘hidden’ or latent vector representation (known as embedding).

In addition to the embedding obtained from the pLMs that captured contextual information on enzyme sequences, forty-one biophysical features were extracted from enzyme sequences, which characterized the biophysical properties of amino acid residues and the whole enzyme sequence. Details of the biophysical features can be found in our previous work (Jiang et al., 2023). In order to represent plastic types, one-hot encoding was used (Yang et al., 2018), and each of the eleven plastic types was one-hot encoded into an 11-bit vector consisting of ten zeros and a one. By concatenating the embedding (a 30-length vector), biophysical features (a 41-length vector), and one-hot encoded plastic types (a 11-length vector), the final feature vector (an 82-length vector) was obtained to represent the raw input (an enzyme/plastic pair) and further used as the input to a supervised ML classification algorithm which is detailed in the next section.

#### 2.2.2 Plastic degradation prediction via classification model

A supervised ML classification algorithm was developed, taking as input the feature vector in section 2.2.1, to perform the binary classification (i.e., degradable/non-degradable). Two classification algorithms were evaluated: (1) multilayer perceptron (MLP) classifier and (2) prototype classifier, with hyperparameters defined in Supplementary Table S3. In addition to the standard MLP classifier, we additionally adopt a prototype-based classification network attempting to improve classification performance upon limited annotated data. In total, six models were developed by the combination of the three pLMs and the two classifiers, referred to as ProtBERT_MLP, ProtBERT_proto, ESM_MLP, ESM_proto, RoBERTa_MLP, and RoBERTa_proto.

The experimental dataset was split into training and testing sets with a ratio of 7:3. Each of the six models, i.e., ProtBERT_MLP, ProtBERT_proto, ESM_MLP, ESM_proto, RoBERTa_MLP, and RoBERTa_proto, was run five times using five random seeds, and model performance results were averaged across the five runs. Model performance was evaluated by a set of widely adopted metrics, including accuracy, precision, recall, and F1 score. The evaluation metrics are defined below, where TP represents true positive, FP represents false positive, TN represents true negative, and FN represents false negative. A higher score of the metrics represents a better performance of the model.

$$\text{Accuracy}=\frac{TP+TN}{TP+FP+TN+FN}$$

$$\text{Precision}=\frac{TP}{TP+FP}$$

$$\text{Recall}=\frac{TP}{TP+FN}$$

$$F1\;\text{score}=\frac{2\cdot Precision\cdot Recall}{Precision+Recall}$$

#### 2.2.3 Confidence and uncertainty estimation

To improve model robustness and reliability, we leveraged ensemble learning to assess the prediction confidence and uncertainty for every enzyme/plastic pair. Let X be the input enzyme/plastic pairs and Y a set of labels indicating the two classes (degradable and non-degradable). A classifier *f*, defined as a function f:X→Y that maps X to Y (Geifman et al., 2018), usually contains a backbone and a classification head (He et al., 2016; Ridnik et al., 2021; Tan and Le, 2019). The backbone extracts features from inputs, and the classification head transforms features into predictions (He et al., 2016). In our study, the classification backbone was replaced by pLM, and an ensemble of N classification heads (N= 25) was used. The *i-*th classification head was denoted by $f_{i}$. Next, for an input enzyme/plastic pair x, prediction confidence can be computed with the mean probability of the predicted class over N (eq. (1)). Similarly, prediction uncertainty was calculated as the standard deviation from the predicted probabilities (eq. (2)). Both confidence and uncertainty values were used to evaluate the prediction output of each enzyme/plastic pair.

(1) $$confidence=\frac{1}{N}\sum_{i=1}^{N}\max\left(\sum_{i=1}^{N}\max\left(f_{i}\left(x\right),1-f_{i}\left(x\right)\right)\;\right)$$

(2) $$uncertainty=\sqrt{\frac{\sum_{i=1}^{N}{\left(\max\left(f_{i}\left(x\right),1-f_{i}\left(x\right)\right)-confidence\right)}^{2}}{N}}$$

During model evaluation on the experimental testing dataset, different thresholds for confidence and uncertainty were tested to filter out low-confidence and high-uncertainty predictions. Specifically, an enzyme/plastic pair with a confidence higher than the confidence threshold and an uncertainty lower than the uncertainty threshold was selected, and otherwise excluded from model evaluation. The evaluation metrics (i.e., accuracy, precision, recall and F1 score detailed in section 2.2.2) were computed over the selected enzyme/plastic pairs at different thresholds. During the analysis on the top-ranked enzyme/plastic pairs, all the input enzyme/plastic pairs were ranked from best to worst by jointly considering the prediction confidence and uncertainty (eq. (3)), and the ranking value was used to generate the heatmaps in section 3.2. Therefore, our approach prioritized high-confidence and low-uncertainty examples while ensuring improved generalization upon unseen data. An enzyme/plastic pair with higher confidence and lower uncertainty had a better ranking and represented a more reliable prediction. During model evaluation on different plastic types, the selected model was run five times on the experimental testing dataset using the same random seeds in section 2.2.2**.** For every enzyme/plastic pair in the experimental testing dataset, confidence and uncertainty values were averaged across the five runs to generate the scatter plot in section 3.2, and the *degradable/non-degradable* prediction was determined by taking a majority vote over the five runs.

(3) $$Ranking=R\left(R\left(confidence\right)+R\left(\frac{1}{uncertainty}\right)\right)$$

Where *R* is the rank function provided by the pandas.DataFrame module in python to compute numerical data ranks.

### 2.3 Sequence similarity calculation

Pairwise sequence alignment for the top list and experimental dataset was conducted by Biopython with the Bio.Blast.Applications module (version 1.81). Computing the percentage of similarity requires dividing identities by the length of sequence with eq. (4) (Yu et al., 2023):

(4) $$Percent\;Similarity\;\left(\%\right)=100\times \frac{Identical\;Residues}{Length\;of\;Smaller\;Sequence}$$

## 3 Results and discussion

### 3.1 Datasets of enzymatic plastic degradation

Two datasets were developed for enzymatic degradation of plastics: (1) the experimental dataset, and (2) the homologous dataset. The experimental dataset included 236 enzyme/plastic pairs, consisting of 171 unique enzyme sequences and 11 types of plastics, including PET, PU, PCL, PLA, poly(ethylene succinate) (PES), poly(ethylene furanoate) (PEF), polyethylene (PE), polyhydroxybutyrate (PHB), polyhydroxyvalerate (PHV), poly(butylene succinate) (PBS), and poly(butylene succinate-*co*-adipate) (PBSA) (Supplementary Fig. S1A). 200 enzyme/plastic pairs were degradable, and 36 enzyme/plastic pairs were non-degradable. The size of the experimental dataset is anticipated to expand as more data from experimental research studies is reported with ongoing efforts in the emerging area of plastic biodegradation. After searching the non-redundant protein sequences database for homologs in NCBI (as described in section 2.1), a homologous dataset consisting of 277,702 enzyme/plastic pairs was created, including 0.1 million unique sequences and 11 plastic types (Supplementary Fig. S1B). These sequences had at least 30% sequence similarity to at least one enzyme in the experimental dataset. The homology-based search results served as the original source of potential plastic-degrading enzymes, as 30% sequence similarity is a rough estimate of similar function (Pearson, 2013). To improve the quality of the homologous dataset, the 277,702 enzyme/plastic pairs were prescreened using the CLEAN tool (Yu et al., 2023). Details of prescreening were provided in section 2.1**.** As a result, 40,853 enzyme/plastic pairs remained in the prescreened homologous dataset (Supplementary Fig. S1C).

### 3.2 Evaluating PEZy-Miner on experimental dataset

The ML-based framework for predicting the degradation ability of an enzyme on a specific type of plastic substrate was designed to integrate a pLM and a classifier along with confidence and uncertainty estimation. In total, six ML models were developed through combination of three pLMs (ProtBERT, ESM-2, and RoBERTa, with details in section 2.2.1) and two classifiers (MLP and prototype classifiers, with details in section 2.2.2). Among the three selected pLMs, BERT-based models (e.g., ProtBERT and RoBERTa) are representative natural language models and could bring great opportunities to protein-related studies (Ferruz et al., 2022), such as protein design (Ferruz et al., 2022, Ferruz and Höcker, 2022), protein folding (Ferruz et al., 2022), antigen-antibody binding (Zhang et al., 2022), and post-translational modifications (Wang et al., 2023). The state-of-the-art ESM-2 model dates to 2023 (Lin et al., 2023) and has substantial advancements in downstream protein prediction tasks, such as classification of antimicrobial peptides (Cordoves-Delgado and García-Jacas, 2024), identification of antigenic determinant region in antigens (Israeli and Louzoun, 2024), and prediction of signal peptide types (Zeng et al., 2023). For the two classifiers used in this study, MLP was selected because the activation functions between layers enabled MLP to tackle complex problems, making MLP one of the most popular neural networks in machine learning area (Blum et al., 2020). However, MLP is prone to generalize poorly with data that is not included in the training dataset, particularly when the size of the dataset is small (Lavine and Blank, 2009). To overcome this limitation, the prototypical network was chosen. Prototypical networks assume that there exists an embedding, or prototype, around which the embedded inputs cluster, so classification can be performed by simply finding the nearest class prototype upon query data (Snell et al., 2017). Such simplicity renders prototypical networks appealing to tasks with limited annotated data (Snell et al., 2017; Li et al., 2019). Each model was run five times using five random seeds and evaluated on the experimental testing dataset.

**Fig. 2:**
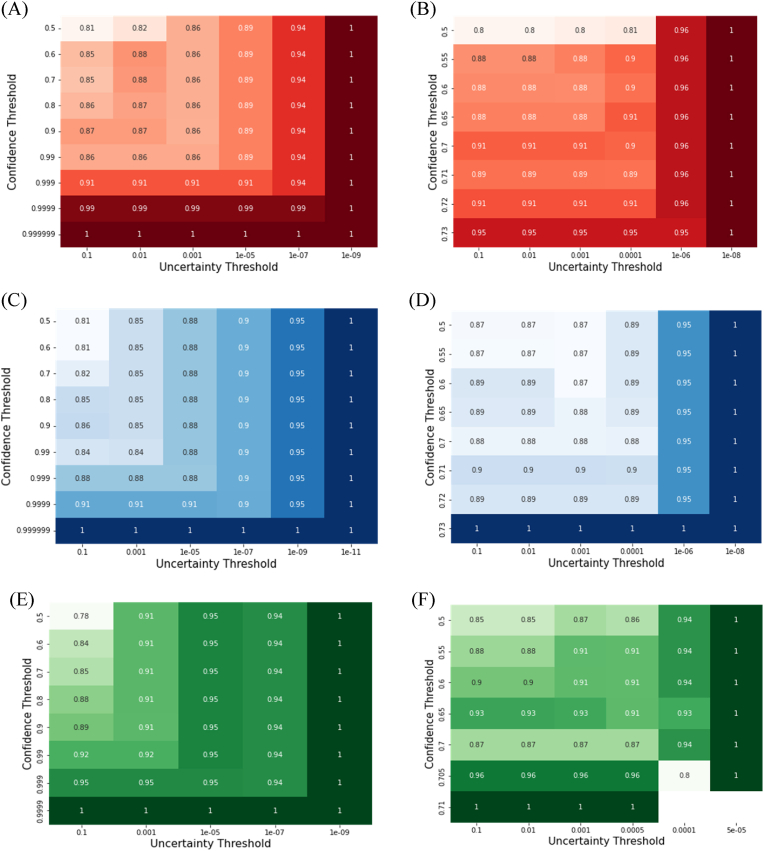
Performance evaluation by accuracy for the ProtBERT_MLP (A), ProtBERT_proto (B), ESM_MLP (C), ESM_proto (D), RoBERTa_MLP (E), and RoBERTa_proto (F) models at different confidence and uncertainty thresholds using the experimental testing dataset. Accuracy values were displayed in every cell on the enzyme/plastic pairs before (top left cell in every subfigure) and after (other cells in every subfigure) filtering by the specified confidence and uncertainty thresholds.

The results based on evaluation metrics of accuracy, precision, recall, and F1 score averaging over five runs were summarized in Fig. 2 and Supplementary Figs. S2–S4, respectively. Before confidence and uncertainty estimation, all the models performed reasonably well with an overall accuracy in the range from 0.783 to 0.873. Further improvement could be achieved by using confidence and uncertainty thresholds to filter out low-confidence and high-uncertainty predictions (with methods detailed in section 2.2.3). As the confidence and uncertainty thresholds became stricter, the prediction accuracy increased and ultimately reached 1 for all the models evaluated on the experimental testing dataset (Fig. 2). Namely, when sorting enzyme/plastic pairs based on prediction confidence and uncertainty, the top-ranked enzyme/plastic pairs could all be correctly classified. The results suggested that the confidence and uncertainty estimation was effective in removing unreliable predictions.

**Fig. 3:**
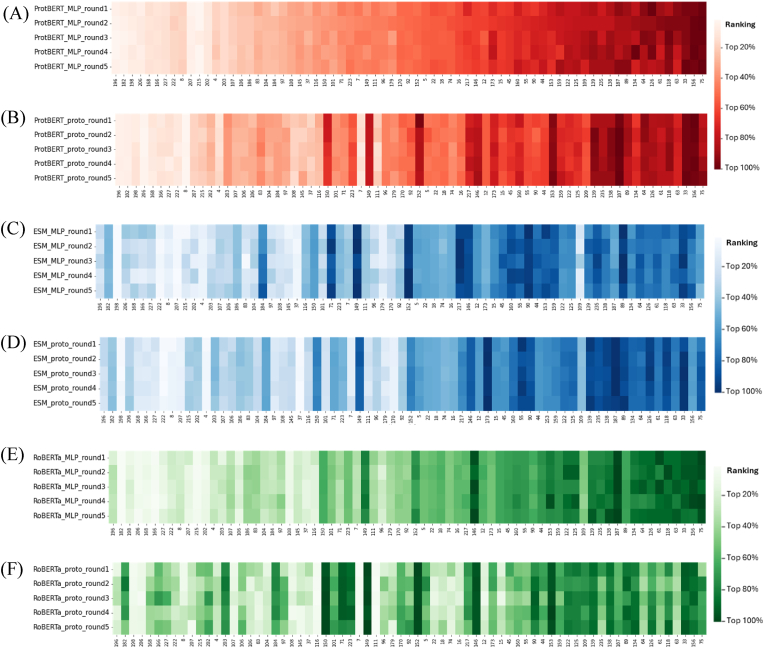
Rankings of the enzyme/plastic pairs in the experimental testing dataset by ProtBERT_MLP (A), ProtBERT_proto (B), ESM_MLP (C), ESM_proto (D), RoBERTa_MLP (E), and RoBERTa_proto (F), with five random seeds for each model. Horizontal axis shows the 71 enzyme/plastic pairs in the experimental testing dataset, each assigned an index for identification purpose. Vertical axis is the five runs for each model. Color gradient represents the ranking values obtained from each run, with light to dark color indicating best to worst ranking of the enzyme/plastic pair in the run. (For interpretation of the references to color in this figure legend, the reader is referred to the Web version of this article.)

Next, we evaluated the six models by analyzing whether the model was able to generate a stable top list. We ran each model five times using five random seeds, ranked the enzyme/plastic pairs in the experimental testing dataset by prediction confidence and uncertainty (with detailed method in section 2.2.3), and analyzed the rankings in the five runs for every model respectively. As denoted by color gradient, the rankings were visualized in Fig. 3 for every enzyme/plastic pair by every model in every run. It was noted that ESM_MLP and RoBERTa_proto showed dispersed color blocks across the five runs, indicating that the model predictions had large variability when changing random seeds. Namely, for each input enzyme/plastic pair, the five rankings obtained from the five runs were divergent when ESM_MLP or RoBERTa_proto was used. By contrast, for the other four models, i.e., ProtBERT_MLP, ProtBERT_proto, ESM_proto, and RoBERTa_MLP, the prediction results within each model were similar across the five runs, suggesting that the four models were able to generate relatively stable predictions respectively (Fig. 3).

We further analyzed the top-ranked enzyme/plastic pairs that were common across the five runs for each model (Table 1). For ProtBERT_MLP, the top 30% enzyme/plastic pairs were mostly the same across all the five runs (20 out of 21 enzyme/plastic pairs), with a prediction accuracy of 1. For ProtBERT_proto, the top 30% enzyme/plastic pairs were mostly the same across all the five runs, with an accuracy of 0.941, while the top 20% enzyme/plastic pairs had a prediction accuracy of 1. The results suggested that ProtBERT_MLP and ProtBERT_proto models were able to generate stable top lists from different runs with random seeds. Similar analysis was performed for ESM- and RoBERTa-based models. Although ESM_proto and RoBERTa_MLP had comparable accuracies to ProtBERT_proto, they returned fewer common enzyme/plastic pairs in the top enzyme/plastic pairs across the five runs. Particularly, for ESM_MLP and RoERTa_proto, the top 30% enzyme/plastic pairs (i.e., the top 21 enzyme/plastic pairs) only had 12 and 11 common enzyme/plastic pairs across the five runs, respectively, and the top 20% pairs (i.e., the top 14 enzyme/plastic pairs) only had 6 and 7 common enzyme/plastic pairs across the five runs, respectively. Overall, the analysis of common enzyme/plastic pairs predicted by each model from different random seeds, for all the experimental testing enzyme/plastic pairs (Fig. 3) and for the top-ranked enzyme/plastic pairs (Table 1) consistently showed that ProtBERT_MLP and ProtBERT_proto outcompeted other models in generating stable top lists of enzyme/plastic pairs. Therefore, ProtBERT_MLP and ProtBERT_proto were selected for subsequent evaluation on homologous dataset, as detailed in section 3.3**.**

**Table 1:** Evaluation of model performance on top list stability for the six models. The number of top-ranked enzyme/plastic pairs that were common across five runs was reported for every model. Accuracy was computed over the top-ranked common enzyme/plastic pairs.

Model	Number of common enzyme/plastic pairs in top 30% or top 21 enzyme/plastic pairs	Accuracy	Number of common enzyme/plastic pairs in top 20% or top 14 enzyme/plastic pairs	Accuracy
ProtBERT_MLP	20	1.000	13	1.000
ProtBERT_proto	17	0.941	12	1.000
ESM_MLP	12	0.833	6	1.000
ESM_proto	20	0.950	9	1.000
RoBERTa_MLP	19	0.947	11	0.909
RoBERTa_proto	11	0.909	7	0.857

The superior performance of ProtBERT-based models compared to RoBERT- or ESM-based models could be attributed to the combined effect of three key factors: (1) the large size of dataset to train ProtBERT, (2) the large model size based on the large number of parameters in ProtBERT, and (3) the appropriate alignment between dataset size and model size. Previous studies showed that increasing dataset size or model size improved language modeling performance (Kaplan et al., 2020; Lin et al., 2023). Meanwhile, it was also suggested that the ratio of training dataset size to model size was an important affecting factor on model performance and that smaller models could outperformed larger models if the ratio of dataset size to model size was more appropriate for small models (Hoffmann et al., 2022). ProtBERT, ESM-2, and RoBERTa pLMs were pre-trained on datasets comprising 216 million (Elnaggar et al., 2022), 43 million (Lin et al., 2023), and 41 thousand sequences, respectively. Meanwhile, ProtBERT was the largest among the three models. ProtBERT had the largest number of parameters, specifically 420 million (Elnaggar et al., 2022), compared to the 150 million parameters of ESM-2 and the 1.7 million parameters of RoBERTa. While the optimal ratio of dataset size to model size is unclear yet (Hoffmann et al., 2022; Kaplan et al., 2020), the large size of dataset and model size could contribute to the superior performance of ProtBERT.

**Fig. 4:**
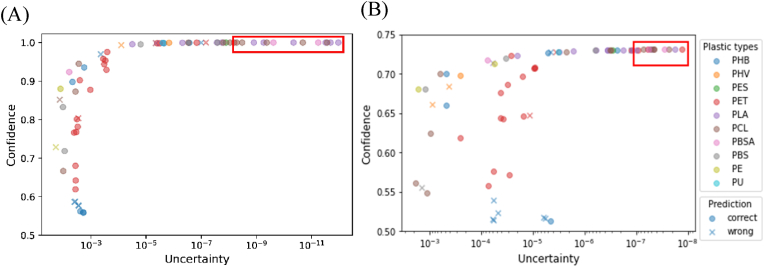
Machine learning predictions on the experimental testing dataset by ProtBERT_MLP (A) and ProtBERT_proto (B). Every marker represented an enzyme/plastic pair in the experimental testing dataset. Different plastic types were indicated by the color of markers. Incorrect predictions were denoted with an 'x'. The top 20% enzyme/plastic pairs were shown in the red window box. (For interpretation of the references to color in this figure legend, the reader is referred to the Web version of this article.)

To account for different plastic types, Fig. 4 investigated model performance using the experimental testing dataset. Inaccurate predictions were distributed in the low confidence and/or high uncertainty region, which contained PET, PHB, PHV and PE. As the confidence and uncertainty thresholds became stricter (from lower left to upper right region), the number of inaccurate predictions decreased to 0 in the top 20% enzyme/plastic pairs boxed in the red window. The majority of the enzyme/plastic pairs correspond to PLA, PCL, and PBSA in the top 20% pairs. These observations indicated that ProtBERT_MLP and ProtBERT_proto could be more effective in identifying enzymes for PLA, PCL, and PBSA degradation compared to other plastics such as PET, PE, PHB, and PHV.

Additionally, we investigated the contribution of three subsets of features—biophysical features, embedding, and plastic features (i.e., one-hot encoded plastic types)—to model performance. By eliminating these feature subsets, their individual contributions were measured by the corresponding decrease in model performance (Tables S4 and S5). The elimination of plastic features led to the most significant drop in accuracy for ProtBERT_MLP compared to other features, indicating that plastic features were most important for ProtBERT_MLP in correctly predicting the degradation of enzyme/plastic pairs. The removal of biophysical features resulted in the largest decrease in precision for ProtBERT_MLP in comparison to other features, suggesting the importance of biophysical features in identifying false positive enzyme/plastic pairs. To be specific, the 4.4% decrease in precision caused by removing biophysical features implied that, among all enzyme/plastic pairs that were predicted to be degradable, 4.4% fewer enzyme/plastic pairs matched ground truths. Embedding had a positive contribution to the performance of ProtBERT_MLP in terms of precision and was the most important feature for ProtBERT_proto across all evaluation metrics. The critical role of embedding revealed the effectiveness of pLMs in the ProtBERT_proto model. Nevertheless, it is noted that most features contributed to model performance in only one or two metrics and had neutral or negative impacts on model performance in other metrics. Future study could focus on model explainability and feature engineering to select the specific features that contribute to the model's success.

### 3.3 Mining plastic degrading enzyme candidates from homologs

With ProtBERT_MLP and ProtBERT_proto models selected through the comprehensive evaluation in section 3.2, we next evaluated the performance of the two models in mining potential plastic degrading enzymes from the prescreened homologous dataset containing 40,853 enzyme/plastic pairs (as detailed in section 2.1). To overcome the challenge of the lack of ground-truth in homologous dataset, we designed a method, referred to as blending assessment, to evaluate the capabilities of the models in shortlisting enzyme candidates. Specifically, we first masked sixty degradable enzyme/plastic pairs in the experimental dataset with experimentally verified ground-truth (referred to as tagged enzyme/plastic pairs) and blended them into the prescreened homologous dataset. Initial concentration of the tagged enzyme/plastic pairs was 0.15% in the prescreened homologous dataset (eq. (5)).

(5) $${\left[conc\right]}_{init}=\frac{number\;of\;tagged\;enzyme/plastic\;pairs\;in\;the\;homologous\;dataset}{number\;of\;enzyme/plastic\;pairs\;in\;the\;homologous\;dataset}=\frac{60}{40853+60}=0.15\%$$

Next, the ML models took as input the prescreened homologous dataset blended with tagged enzyme/plastic pairs, predicted degradability on all the input enzyme/plastic pairs, and identified top-ranked enzyme/plastic pairs with confidence and uncertainty estimation. Each model was run five times, and the top-ranked enzyme/plastic pairs (e.g., top 0.5%–1%) which were common across the five runs were identified as top list from the model prediction. In the obtained top lists from the two models, ProtBERT_MLP and ProtBERT_proto, the concentration of tagged enzyme/plastic pairs was calculated (eq. (6) and eq. (7)).

(6) $${\left[conc\right]}_{final}=\frac{number\;of\;tagged\;pairs\;in\;the\;top\;list}{number\;of\;pairs\;in\;the\;top\;list}$$

(7) $$Concentration\;factor=\frac{{\left[conc\right]}_{init}}{{\left[conc\right]}_{final}}$$

The results of blending assessment, as summarized in Table 2, suggested the effectiveness of both ProtBERT_MLP and ProtBERT_proto in shortlisting plastic degrading enzyme candidates. For ProtBERT_MLP model, the concentration of tagged enzyme/plastic pairs increased from the original 0.15%–4.90% and 2.64% in the top 0.5% and 1% lists, respectively, meaning that the model was able to concentrate the enzyme/plastic pairs with known degradability by 18–33 times. Similarly, for ProtBERT_proto, the tagged enzyme/plastic pairs were concentrated 14–25 times in the top lists. The predictions for all the tagged enzyme/plastic pairs shortlisted in the top lists were degradable, which matched the ground-truth. Considering the case of random sampling of a subset of enzyme/plastic pairs (e.g. 0.5% or 1%) from the total homologous and tagged enzyme/plastic pairs in the dataset, the concentration of tagged pairs would remain unchanged after sampling (i.e., as the initial 0.15%). The substantial enrichment of degradable tagged enzyme/plastic pairs in the predicted top lists suggested that our models were effective in identifying promising enzyme candidates with high plastic degradation potential. Notably, among the top lists, the tagged enzyme/plastic pairs consisted of a small portion, while the majority were enzyme/plastic pairs from homologous dataset. The prediction of the enzyme/plastic pairs from homologous dataset on the same top list as the experimentally verified ones provided confidence that these unexplored enzymes could be promising candidates for the target plastic degradation.

**Table 2:** Blending assessment on ProtBERT_MLP and ProtBERT_proto. In the top 0.5% or top 1% list identified by each model, the number of enzyme/plastic pairs and the number of tagged enzyme/plastic pairs were reported. [conc]_final_ represented the final concentration of the tagged pairs in the top list. The concentration factor was computed based on the initial and final concentrations.

Model	Top rank	Number of enzyme/plastic pairs in the top list^a^	Number of tagged enzyme/plastic pairs in the top list^b^	[conc]_final_	Concentration factor
ProtBERT_MLP	Top 0.5%	102	5	4.90 %	33
	Top 1%	227	6	2.64 %	18
ProtBERT_proto	Top 0.5%	80	3	3.75%	25
	Top 1%	187	4	2.14 %	14
ProtBERT_MLP and ProtBERT_proto	Top 0.5%	31	1	3.23 %	22
	Top 1%	78	3	3.85 %	26

a All pairs in the top list were predicted to be degradable.

b The predictions matched the ground-truth, and both were degradable for the tagged pairs.

**Fig. 5:**
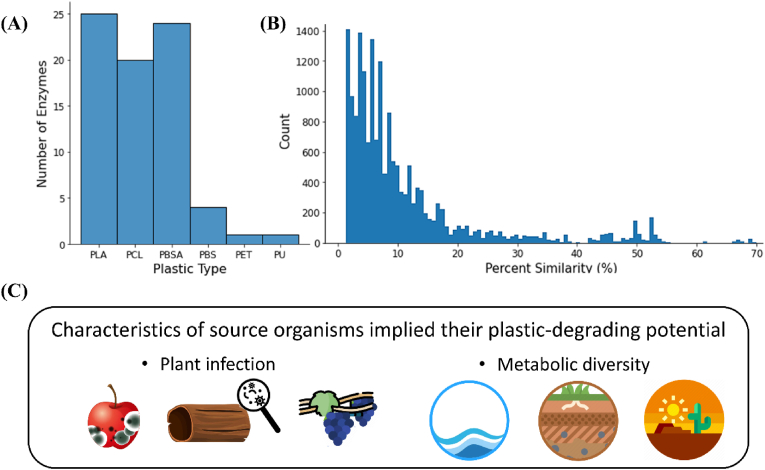
Biological insights into enzyme candidates in the top list. (A) Distribution of enzymes involved in degradation of different plastic types in the top list, identified by the combination of ProtBERT_MLP and ProtBERT_proto models. (B) Distribution of pairwise sequence similarities between the top list and the experimental dataset. (C) Illustration of the associated plants and living environments of the source organisms of enzyme candidates.

To obtain the most promising enzyme candidates, we further explored the top list prediction by combining ProtBERT_MLP and ProtBERT_proto models. Specifically, common enzyme/plastic pairs predicted across the ten runs of the two models at a specified top list threshold were identified. In the top 1% list from model prediction, 78 common enzyme/plastic pairs were found in both model predictions (Table 2). There were 3 tagged enzyme/plastic pairs and 75 homologous enzyme/plastic pairs, including 27 unique homologs and 6 plastic types spanning PLA, PCL, PBSA, PBS, PET and PU (Fig. 5A). Since these 75 enzyme/plastic pairs were consistently ranked within the top 1% by both models, they could serve as a list of promising candidates with prediction reliability for future research efforts in discovering novel plastic degrading enzymes.

### 3.4 Biological insights into the top list

With the 75 enzyme/plastic pairs identified as a list of promising candidates by PEZy-Miner, we collected detailed information from NCBI to gain biological insights (Fig. 5C). First, the majority of the enzymes were annotated as cutinases by NCBI, which aligned well with established knowledge that cutinases were active on polyesters because they lack a lid structure and that the exposed active site is essential for the interaction with plastic substrates (Chen et al., 2020; Wei and Zimmermann, 2017b). Second, we found most sequences originated from fungi which were associated with plant diseases, fruit rot, and leaf penetration. For example, several candidates were from the *Fusarium* genus, and species in the genus have been reported to be the causal agents of apple rot and pea root rot (Coleman et al., 2009; Petreš et al., 2023). Other than *Fuscarium*, we also discovered candidates produced by fungi from *Dactylonectria* and *Phaeomoniella*, the genera associated with destructive trunk diseases on grapevine and/or root rot of olive trees (Mesny et al., 2021; Morales-Cruz et al., 2015). This observation was consistent with existing knowledge that some microorganisms known for degrading plant biomass have also demonstrated the capability in degrading plastics, as both substrates had similar linkages, hydrophobic surfaces, and high-molecular-weight forms (Chen et al., 2020). Third, some source organisms of the enzyme candidates were reported to be able to colonize a wide range of environments. *Fuscarium* fungi were found in soil, deserts, coastal zones, painted caves and nuclear reactors (Dupont et al., 2007; Mandeel, 1996; Wainwright et al., 1994; Zhdanova et al., 2000). The abilities to adapt to various environments reflect their metabolic diversity (Coleman et al., 2009), where metabolic pathways for plastic utilization could also exist. Notably, two species of *Fusarium* were reported to be plastic degraders, *F. oxysporum* (Nimchua et al., 2007), and *F. vanettenii* (formerly referred to as *F. solani*) (Silva et al., 2005; Yoshida et al., 2016).

Additionally, pairwise similarity between the top list and the experimental dataset was computed, with calculation detailed in section 2.3**.** In general, the top list was dissimilar from well-characterized enzymes (Fig. 5B). Out of the 75 enzyme/plastic pairs, only 20 enzyme/plastic pairs shared sequence similarities of over 60% with at least one enzyme in the experimental dataset. The highest similarity was 70%, which was found between a well-studied *Aspergillus oryzae* cutinase (GenBank accession number: P52956) that exhibited hydrolytic activities on PLA, PBS, and PBSA (Liu et al., 2009; Maeda et al., 2005), and a putative protein (GenBank accession number: KAF9893366.1) from the genome sequencing of *Aspergillus nanangensis*, a fungal species known for producing antiviral molecules (Lacey et al., 2019). The biological insights gained from the analysis of the enzyme candidates provided a foundational understanding of their potential in plastic degradation.

## 4 Conclusions

In this study, we designed an innovative ML approach, PEZy-Miner, to discover promising enzyme candidates capable of degrading plastics of interest. PEZy-Miner takes as input a custom dataset consisting of enzyme/plastic pairs, runs five times, and returns top-ranked enzyme/plastic pairs common across the five runs as the candidate list. Three options are provided for the pLM module and the classification module: the ProtBERT_MLP model, the ProtBERT_proto model, and the combination of the two models. An adjustable percentile is available in the confidence and uncertainty estimation module to control the number of enzyme/plastic pairs in the top list according to the research needs.

PEZy-Miner was comprehensively evaluated using experimentally verified enzyme/plastic pairs with ground-truth. The framework was accurate in identifying degradable enzyme/plastic pairs and powerful in shortlisting a given dataset of unknown enzyme/plastic pairs. We envision the use of PEZy-Miner in recommending enzyme candidates before experimental research, thereby potentially reducing the time, labor, and expenses of experimentation. It is noted that building of the homologous dataset described in this study is not the only way to generate the source for plastic-degrading enzyme mining. Users of PEZy-Miner may build their own customized dataset for the discovery of potential enzymes for degradation of plastics of interest. Our ongoing work is currently focused on experimental testing of the 75 enzyme/plastic pairs discovered in this study.

## Funding

This work was supported by the 10.13039/100000001National Science Foundation [Grant CNS-1831669]; and the 10.13039/100005302University of Illinois Urbana−Champaign.

## CRediT authorship contribution statement

**Renjing Jiang:** Writing – review & editing, Writing – original draft, Methodology, Investigation, Formal analysis, Conceptualization. **Zhenrui Yue:** Writing – original draft, Methodology, Investigation, Formal analysis, Conceptualization. **Lanyu Shang:** Methodology, Formal analysis. **Dong Wang:** Writing – review & editing, Supervision, Project administration, Methodology, Investigation, Funding acquisition, Formal analysis, Conceptualization. **Na Wei:** Writing – review & editing, Supervision, Resources, Project administration, Methodology, Investigation, Funding acquisition, Conceptualization.

## Declaration of competing interest

The authors declare that they have no known competing financial interests or personal relationships that could have appeared to influence the work reported in this paper.

## Data Availability

Data will be made available on request.

## References

[bib1] Altschul S.F., Gish W., Miller W., Myers E.W., Lipman D.J. (1990). Basic local alignment search tool. J. Mol. Biol..

[bib2] Bardají D.K.R., Furlan J.P.R., Stehling E.G. (2019). Isolation of a polyethylene degrading *Paenibacillus* sp. from a landfill in Brazil. Arch. Microbiol..

[bib3] Blum A., Hopcroft J., Kannan R. (2020).

[bib4] Buchholz P.C.F., Feuerriegel G., Zhang H., Perez-Garcia P., Nover L.-L., Chow J., Pleiss J. (2022). Plastics degradation by hydrolytic enzymes: the plastics-active enzymes database—PAZy. Proteins: Struct., Funct., Bioinf..

[bib5] Chang X., Xue Y., Li J., Zou L., Tang M. (2020). Potential health impact of environmental micro- and nanoplastics pollution. J. Appl. Toxicol..

[bib6] Chen C.-C., Dai L., Ma L., Guo R.-T. (2020). Enzymatic degradation of plant biomass and synthetic polymers. Nat. Rev. Chem.

[bib7] Chowdhary K.R., Chowdhary K.R. (2020). Fundamentals of Artificial Intelligence.

[bib8] Coleman J.J., Rounsley S.D., Rodriguez-Carres M., Kuo A., Wasmann C.C., Grimwood J., VanEtten H.D. (2009). The genome of nectria haematococca: contribution of supernumerary chromosomes to gene expansion. PLoS Genet..

[bib9] Cordoves-Delgado G., García-Jacas C.R. (2024). Predicting antimicrobial peptides using ESMFold-predicted structures and ESM-2-based amino acid features with graph deep learning. J. Chem. Inf. Model..

[bib10] Danso D., Schmeisser C., Chow J., Zimmermann W., Wei R., Leggewie C., Streit W.R. (2018). New insights into the function and global distribution of polyethylene terephthalate (PET)-Degrading bacteria and enzymes in marine and terrestrial metagenomes. Appl. Environ. Microbiol..

[bib11] Domingos P. (2012). A few useful things to know about machine learning. Commun. ACM.

[bib12] Dupont J., Jacquet C., Dennetiere B., Lacoste S., Bousta F., Orial G., Roquebert M.-F. (2007). Invasion of the French Paleolithic painted cave of Lascaux by members of the Fusarium solani species complex. Mycologia.

[bib13] Elnaggar A., Heinzinger M., Dallago C., Rehawi G., Wang Y., Jones L., Rost B. (2022). ProtTrans: toward understanding the language of life through self-supervised learning. IEEE Trans. Pattern Anal. Mach. Intell..

[bib14] Ferruz N., Höcker B. (2022). Controllable protein design with language models. Nat. Mach. Intell..

[bib15] Ferruz N., Schmidt S., Höcker B. (2022). ProtGPT2 is a deep unsupervised language model for protein design. Nat. Commun..

[bib16] Geifman Y., Uziel G., El-Yaniv R. (2018).

[bib17] Hajighasemi M., Tchigvintsev A., Nocek B., Flick R., Popovic A., Hai T., Yakunin A.F. (2018). Screening and characterization of novel polyesterases from environmental metagenomes with high hydrolytic activity against synthetic polyesters. Environ. Sci. Technol..

[bib18] He K., Zhang X., Ren S., Sun J. (2016). Paper Presented at the 2016 IEEE Conference on Computer Vision and Pattern Recognition (CVPR).

[bib19] Hie B.L., Shanker V.R., Xu D., Bruun T.U.J., Weidenbacher P.A., Tang S., Kim P.S. (2024). Efficient evolution of human antibodies from general protein language models. Nat. Biotechnol..

[bib20] Hoffmann J., Borgeaud S., Mensch A., Buchatskaya E., Cai T., Rutherford E., Clark A. (2022).

[bib21] Israeli S., Louzoun Y. (2024). Single-residue linear and conformational B cell epitopes prediction using random and ESM-2 based projections. Briefings Bioinf..

[bib22] Jeon H.J., Kim M.N. (2015). Functional analysis of alkane hydroxylase system derived from Pseudomonas aeruginosa E7 for low molecular weight polyethylene biodegradation. Int. Biodeterior. Biodegrad..

[bib23] Jeon H.J., Kim M.N. (2016). Comparison of the functional characterization between alkane monooxygenases for low-molecular-weight polyethylene biodegradation. Int. Biodeterior. Biodegrad..

[bib24] Jiang R., Shang L., Wang R., Wang D., Wei N. (2023). Machine learning based prediction of enzymatic degradation of plastics using encoded protein sequence and effective feature representation. Environ. Sci. Technol. Lett..

[bib25] Kaplan J., McCandlish S., Henighan T., Brown T.B., Chess B., Child R., Amodei D. (2020).

[bib26] Kim D.-W., Ahn J.-H., Cha C.-J. (2022). Biodegradation of plastics: mining of plastic-degrading microorganisms and enzymes using metagenomics approaches. J. Microbiol..

[bib27] Lacey H.J., Gilchrist C.L.M., Crombie A., Kalaitzis J.A., Vuong D., Rutledge P.J., Piggott A.M. (2019). Nanangenines: drimane sesquiterpenoids as the dominant metabolite cohort of a novel Australian fungus, Aspergillus nanangensis. Beilstein J. Org. Chem..

[bib28] Lavine B.K., Blank T.R. (2009). Feed-forward neural networks. Comprehensive Chemometrics.

[bib29] Li X., Yu L., Cao J., Chang D., Ma Z., Liu N. (2019). Asia-Pacific Signal and Information Processing Association Annual Summit and Conference.

[bib30] Lin Z., Akin H., Rao R., Hie B., Zhu Z., Lu W., Rives A. (2023). Evolutionary-scale prediction of atomic-level protein structure with a language model. Science.

[bib31] Liu X., Lu D., Zhang A., Liu Q., Jiang G. (2022). Data-driven machine learning in environmental pollution: gains and problems. Environ. Sci. Technol..

[bib32] Liu Y., Ott M., Goyal N., Du J., Joshi M., Chen D., Stoyanov V. (2019).

[bib33] Liu Z., Gosser Y., Baker P.J., Ravee Y., Lu Z., Alemu G., Montclare J.K. (2009). Structural and functional studies of Aspergillus oryzae cutinase: enhanced thermostability and hydrolytic activity of synthetic ester and polyester degradation. J. Am. Chem. Soc..

[bib34] Lu H., Diaz D.J., Czarnecki N.J., Zhu C., Kim W., Shroff R., Alper H.S. (2022). Machine learning-aided engineering of hydrolases for PET depolymerization. Nature.

[bib35] Madani A., Krause B., Greene E.R., Subramanian S., Mohr B.P., Holton J.M. (2023). Large language models generate functional protein sequences across diverse families. Nat. Biotechnol..

[bib36] Maeda H., Yamagata Y., Abe K., Hasegawa F., Machida M., Ishioka R., Nakajima T. (2005). Purification and characterization of a biodegradable plastic-degrading enzyme from Aspergillus oryzae. Appl. Microbiol. Biotechnol..

[bib37] Mandeel Q. (1996). Prevalence of Fusarium Species in Various Soil Groups Using Several Isolation Techniques.

[bib39] Mesny F., Miyauchi S., Thiergart T., Pickel B., Atanasova L., Karlsson M., Hacquard S. (2021). Genetic determinants of endophytism in the Arabidopsis root mycobiome. Nat. Commun..

[bib40] Mitchell T.M., Mitchell T.M. (1997).

[bib41] Morales-Cruz A., Amrine K.C.H., Blanco-Ulate B., Lawrence D.P., Travadon R., Rolshausen P.E., Cantu D. (2015). Distinctive expansion of gene families associated with plant cell wall degradation, secondary metabolism, and nutrient uptake in the genomes of grapevine trunk pathogens. BMC Genom..

[bib42] Nimchua T., Punnapayak H., Zimmermann W. (2007). Comparison of the hydrolysis of polyethylene terephthalate fibers by a hydrolase from Fusarium oxysporum LCH I and Fusarium solani f. sp. pisi. Biotechnol. J..

[bib43] Oh Y.R., Jang Y.A., Song J.K., Eom G.T. (2022). Efficient enzymatic depolymerization of polycaprolactone into 6-hydroxyhexanoic acid by optimizing reaction conditions and microbial conversion of 6-hydroxyhexanoic acid into adipic acid for eco-friendly upcycling of polycaprolactone. Biochem. Eng. J..

[bib44] Pearson W.R. (2013). An introduction to sequence similarity (“Homology”) searching. Current Protocols in Bioinformatics.

[bib45] Petreš M., Hrustić J., Vučinić N., Ma L.-J., Ayhan Dilay H., Grahovac M. (2023). Genome sequence resource of Fusarium graminearum TaB10 and Fusarium avenaceum KA13, causal agents of stored apple rot. MPMI (Mol. Plant-Microbe Interact.).

[bib47] Ridnik T., Lawen H., Noy A., Ben E., Sharir B.G., Friedman I. (2021). Paper Presented at the 2021 IEEE Winter Conference on Applications of Computer Vision (WACV).

[bib48] Rives A., Meier J., Sercu T., Goyal S., Lin Z., Liu J. (2021). Biological structure and function emerge from scaling unsupervised learning to 250 million protein sequences. Proc. Natl. Acad. Sci. USA.

[bib49] Rochman C.M., Browne M.A., Halpern B.S., Hentschel B.T., Hoh E., Karapanagioti H.K., Thompson R.C. (2013). Classify plastic waste as hazardous. Nature.

[bib50] Roohi Bano K., Kuddus M., Zaheer R.M., Zia Q., Khan F.M., Aliev G. (2017). Microbial enzymatic degradation of biodegradable plastics. Curr. Pharmaceut. Biotechnol..

[bib51] Ruffolo J.A., Madani A. (2024). Designing proteins with language models. Nat. Biotechnol..

[bib52] Sanluis-Verdes A., Colomer-Vidal P., Rodriguez-Ventura F., Bello-Villarino M., Spínola-Amilibia M., Ruiz-Lopez E. (2022). Wax worm saliva and the enzymes therein are the key to polyethylene degradation by Galleria mellonella. Nat. Commun..

[bib53] Shin J.-E., Riesselman A.J., Kollasch A.W., McMahon C., Simon E., Sander C., Marks D.S. (2021). Protein design and variant prediction using autoregressive generative models. Nat. Commun..

[bib54] Silva C.M., Carneiro F., O'Neill A., Fonseca L.P., Cabral J.S., Guebitz G., Cavaco‐Paulo A. (2005). Cutinase—a new tool for biomodification of synthetic fibers. J. Polym. Sci. Polym. Chem..

[bib55] Smith M., Love D.C., Rochman C.M., Neff R.A. (2018). Microplastics in seafood and the implications for human health. Current Environmental Health Reports.

[bib56] Snell J., Swersky K., Zemel R. (2017). Prototypical networks for few-shot learning. Adv. Neural Inf. Process. Syst..

[bib57] Sourkouni G., Jeremić S., Kalogirou C., Höfft O., Nenadovic M., Jankovic V. (2023). Study of PLA pre-treatment, enzymatic and model-compost degradation, and valorization of degradation products to bacterial nanocellulose. World J. Microbiol. Biotechnol..

[bib58] Sturmberger L., Wallace P.W., Glieder A., Birner-Gruenberger R. (2016). Synergism of proteomics and mRNA sequencing for enzyme discovery. J. Biotechnol..

[bib59] Tan M., Le Q.V. (2019).

[bib60] Taniguchi I., Yoshida S., Hiraga K., Miyamoto K., Kimura Y., Oda K. (2019). Biodegradation of PET: current status and application aspects. ACS Catal..

[bib61] Temporiti M.E.E., Nicola L., Nielsen E., Tosi S. (2022). Fungal enzymes involved in plastics biodegradation. Microorganisms.

[bib62] Tian W., Skolnick J. (2003). How well is enzyme function conserved as a function of pairwise sequence identity?. J. Mol. Biol..

[bib63] Viljakainen V.R., Hug L.A. (2021). New approaches for the characterization of plastic-associated microbial communities and the discovery of plastic-degrading microorganisms and enzymes. Comput. Struct. Biotechnol. J..

[bib64] Viljakainen V.R., Hug L.A. (2021). The phylogenetic and global distribution of bacterial polyhydroxyalkanoate bioplastic-degrading genes. Environ. Microbiol..

[bib65] Wainwright M., Ali T.A., Killham K. (1994). Anaerobic growth of fungal mycelium from soil particles onto nutrient-free silica gel. Mycol. Res..

[bib66] Wang X., Ding Z., Wang R., Lin X. (2023). Deepro-Glu: combination of convolutional neural network and Bi-LSTM models using ProtBert and handcrafted features to identify lysine glutarylation sites. Briefings Bioinf..

[bib67] Wei R., Zimmermann W. (2017). Biocatalysis as a green route for recycling the recalcitrant plastic polyethylene terephthalate. Microb. Biotechnol..

[bib68] Wei R., Zimmermann W. (2017). Microbial enzymes for the recycling of recalcitrant petroleum-based plastics: how far are we?. Microb. Biotechnol..

[bib69] Yang K.K., Wu Z., Bedbrook C.N., Arnold F.H. (2018). Learned protein embeddings for machine learning. Bioinformatics.

[bib70] Yoshida S., Hiraga K., Takehana T., Taniguchi I., Yamaji H., Maeda Y., Oda K. (2016). A bacterium that degrades and assimilates poly(ethylene terephthalate). Science.

[bib71] Yu T., Cui H., Li J.C., Luo Y., Jiang G., Zhao H. (2023). Enzyme function prediction using contrastive learning. Science.

[bib72] Zeng S., Wang D., Dong X. (2023).

[bib73] Zhang Y., Zhu G., Li K., Li F., Huang L., Duan M., Zhou F. (2022). HLAB: learning the BiLSTM features from the ProtBert-encoded proteins for the class I HLA-peptide binding prediction. Briefings Bioinf..

[bib74] Zhdanova N.N., Zakharchenko V.A., Vember V.V., Nakonechnaya L.T. (2000). Fungi from Chernobyl: mycobiota of the inner regions of the containment structures of the damaged nuclear reactor. Mycol. Res..

[bib75] Zhu B., Wang D., Wei N. (2022). Enzyme discovery and engineering for sustainable plastic recycling. Trends Biotechnol..

[bib76] Zrimec J., Kokina M., Jonasson S., Zorrilla F., Zelezniak A. (2021). Plastic-degrading potential across the global microbiome correlates with recent pollution trends. mBio.

