# Supplementary material for: PEZy-miner: An artificial intelligence driven approach for the discovery of plastic-degrading enzyme candidates
Source: Metab Eng Commun. 2024 Sep 5;19:e00248. doi: 10.1016/j.mec.2024.e00248 (PMC11414552; doi:10.1016/j.mec.2024.e00248)
Supplement: Multimedia component 1 [file mmc1.docx]

# Supplementary Information

**PEZy-Miner: An artificial intelligence driven approach for the discovery of Plastic-degrading EnZYme candidates**

Renjing Jiang^1^, Zhenrui Yue^2^, Lanyu Shang^2^, Dong Wang*^2^, and Na Wei*^1^

1 Department of Civil and Environmental Engineering, University of Illinois Urbana-Champaign, Urbana, Illinois, 61801, United States

2 School of Information Sciences, University of Illinois Urbana-Champaign, Champaign, Illinois, 61820, United States

* Corresponding Author: Na Wei, E-mail: [nawei2@illinois.edu](mailto:nawei2@illinois.edu), Dong Wang: E-mail: [dwang24@illinois.edu](mailto:dwang24@illinois.edu)

**Table S1.** Algorithm parameters used in Basic Local Alignment Search Tool (BLAST) searches.

| **Parameter Name** | **Parameter Value** |
| --- | --- |
| Database | Non-redundant protein sequences (nr) |
| Algorithm | BLASTP (protein-protein BLAST) |
| Max target sequences | 5,000 |
| Expect threshold | 0.05 |
| Word size | 5 |
| Matrix | BLOSUM62 |
| Gap Costs | Existence: 11 Extension: 1 |
| Compositional adjustments | Conditional compositional score matrix adjustment |

**Table S2.** Configurations for pre-training the protein language models (pLMs).

| **pLM** | **Dataset** | **Number of Layers** | **Number of Parameters** | **Reference** |
| --- | --- | --- | --- | --- |
| ProtBERT | UniRef100 | 30 | 420M | Elnaggar et al.^34^ |
| ESM-2* | UniRef50 | 30 | 150M | Lin et al.^35^ |
| RoBERTa | The homologous dataset in this study | 8 | 1.7M | Lin et al.^36^ |

* ESM-2 released six models, with the number of parameters varying from 8 million to 15 billion. In this study, we used the ESM2_t30_150M_UR50D model.

**Table S3.** Hyperparameters for the multilayer perceptron (MLP) and prototype classifier.

| **Hyperparameter** | **MLP** | **Prototype** |
| --- | --- | --- |
| Max iteration | 500 | 100 |
| Batch size | 32 | 128 |
| Number of hidden layers | 2 | 2 |
| L2 penalty parameter | 1e-5 | 1e-2 |
| Learning rate | 1e-3 | 1e-4 |
| Optimizer | Adam | Adam |
| Activation | ReLU | None |

**Table S4.** Contribution of plastic features, biophysical features, and embedding to the performance of ProtBERT_MLP measured by accuracy, precision, recall, and F1 score.

| **Included features** | **Accuracy** | **Precision** | **Recall** | **F1 score** |
| --- | --- | --- | --- | --- |
| All features | 0.806 | 0.897 | 0.870 | 0.883 |
| Without plastic features | 0.790 | 0.862 | 0.910 | 0.884 |
| Without biophysical features | 0.831 | 0.853 | 0.967 | 0.906 |
| Without embedding | 0.808 | 0.893 | 0.878 | 0.885 |

**Table S5.** Contribution of plastic features, biophysical features, and embedding to the performance of ProtBERT_proto measured by accuracy, precision, recall, and F1 score.

| **Included features** | **Accuracy** | **Precision** | **Recall** | **F1 score** |
| --- | --- | --- | --- | --- |
| All features | 0.797 | 0.883 | 0.877 | 0.880 |
| Without plastic features | 0.785 | 0.863 | 0.887 | 0.875 |
| Without biophysical features | 0.831 | 0.853 | 0.967 | 0.906 |
| Without embedding | 0.610 | 0.852 | 0.647 | 0.698 |

(A)


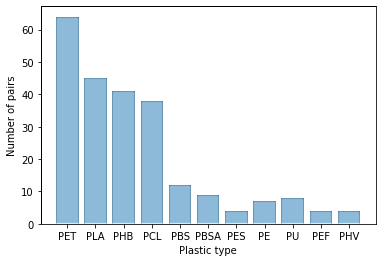


(B)


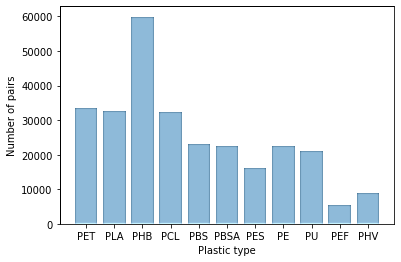


(C)


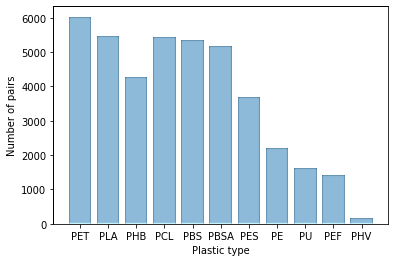


**Figure S1.** Distribution of the number of enzymes relevant to degradation of different types of plastics. (A) Distribution of enzymes involved in degradation of different plastic types in the experimental dataset. (B) Distribution of sequences in the homologous dataset. (C) Distribution of sequences in the prescreened homologous dataset.


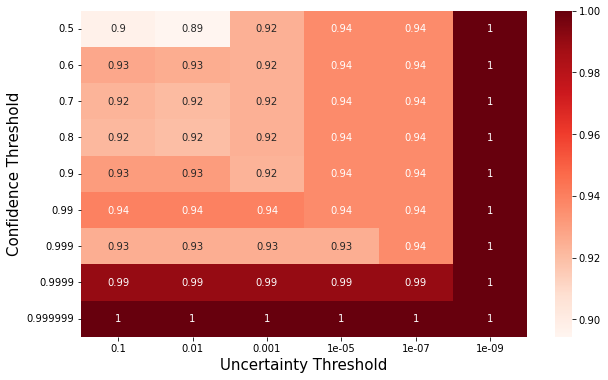

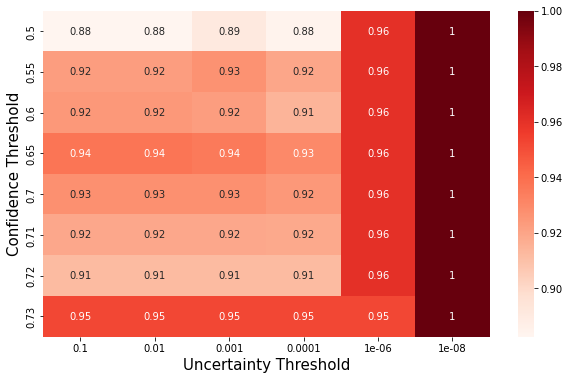


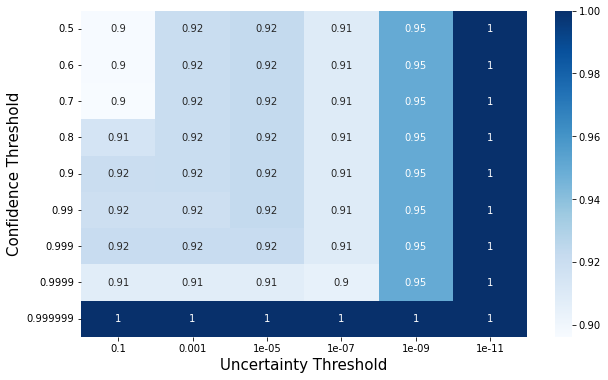

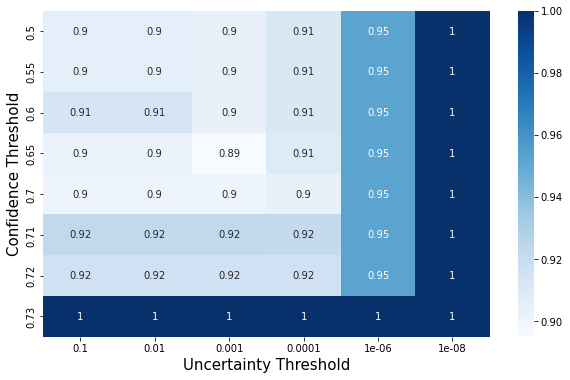


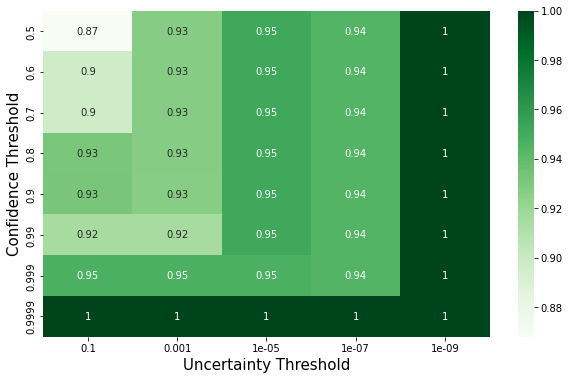

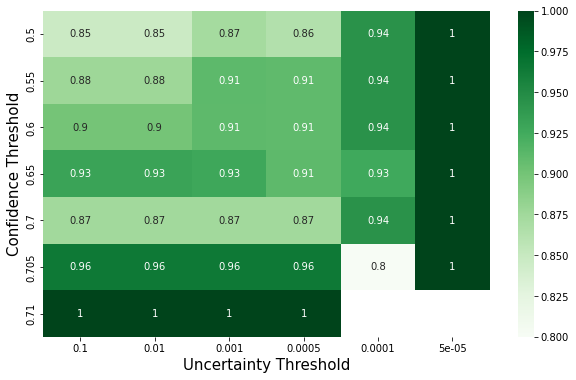


**Figure S2.** Performance evaluation by precision for the ProtBERT_MLP (A), ProtBERT_proto (B), ESM_MLP (C), ESM_proto (D), RoBERTa_MLP (E), and RoBERTa_proto (F) models at different confidence and uncertainty thresholds using the experimental testing dataset. Precision values were displayed in every cell on the enzyme/plastic pairs before (top left cell in every plot) and after (other cells in every plot) filtering by the specified confidence and uncertainty thresholds.


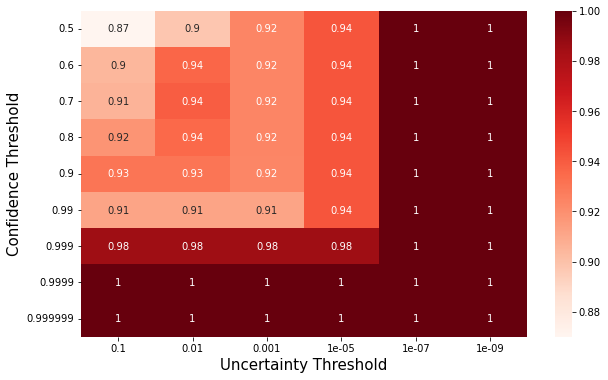

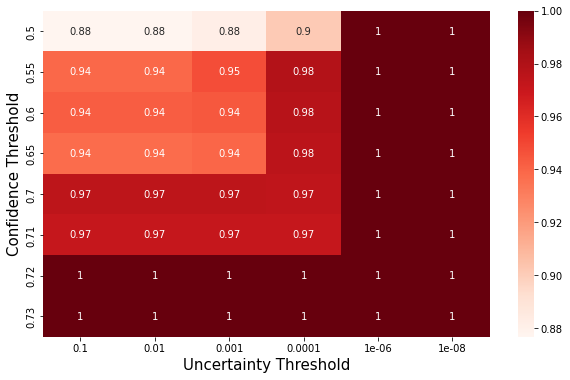


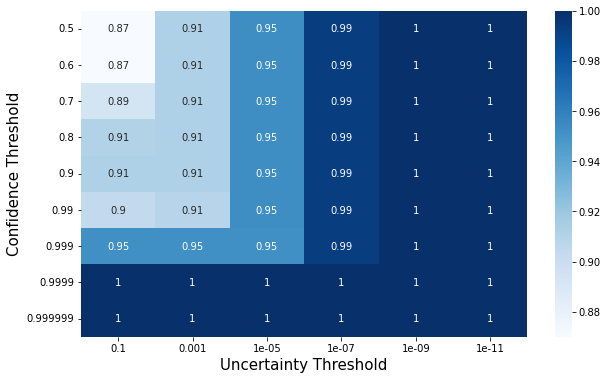

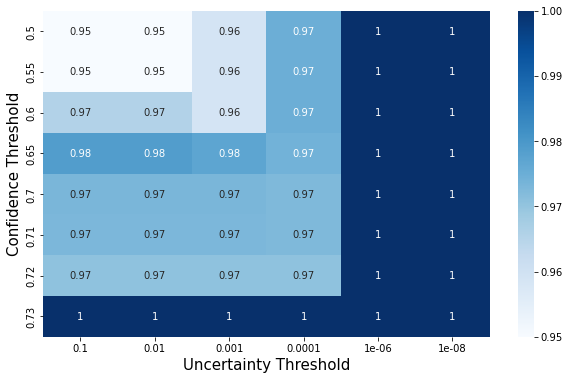


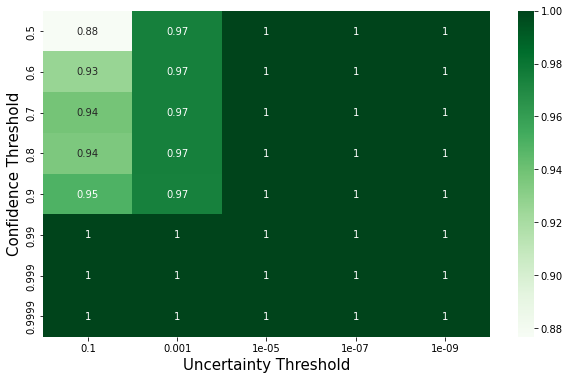

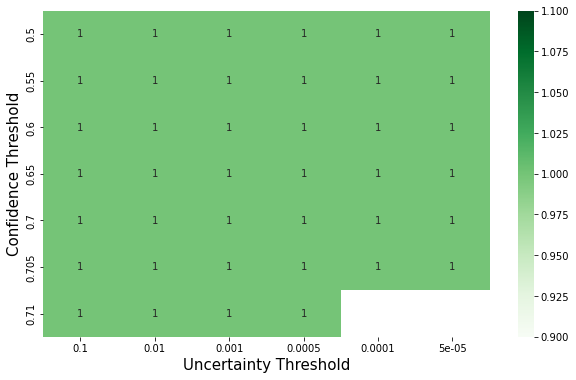


**Figure S3.** Performance evaluation by recall for the ProtBERT_MLP (A), ProtBERT_proto (B), ESM_MLP (C), ESM_proto (D), RoBERTa_MLP (E), and RoBERTa_proto (F) models at different confidence and uncertainty thresholds using the experimental testing dataset. Recall values were displayed in every cell on the enzyme/plastic pairs before (top left cell in every plot) and after (other cells in every plot) filtering by the specified confidence and uncertainty thresholds.


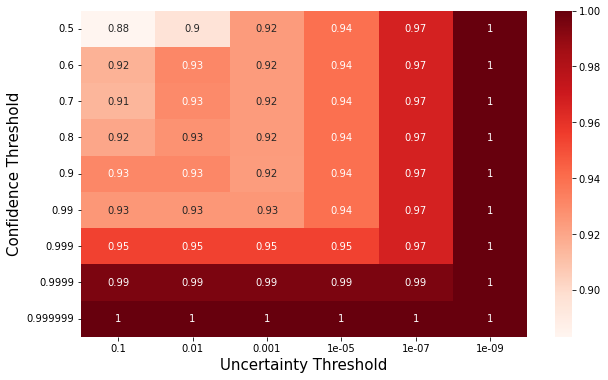

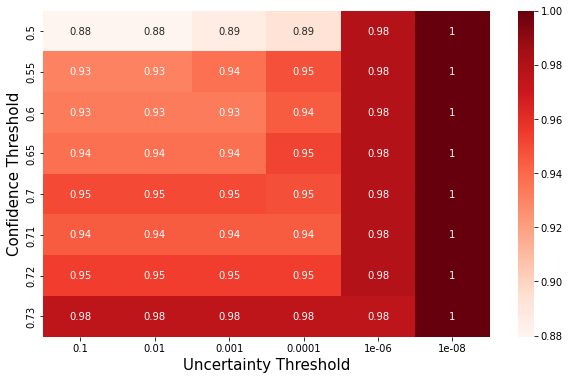


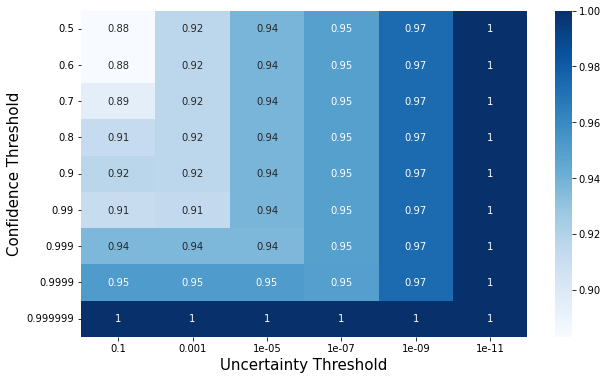

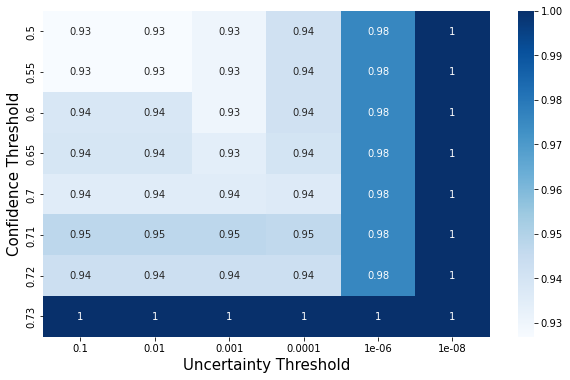


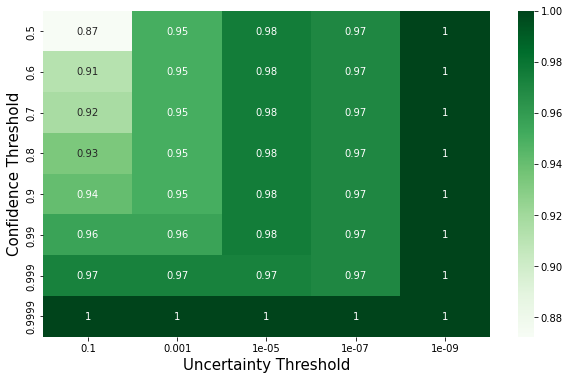

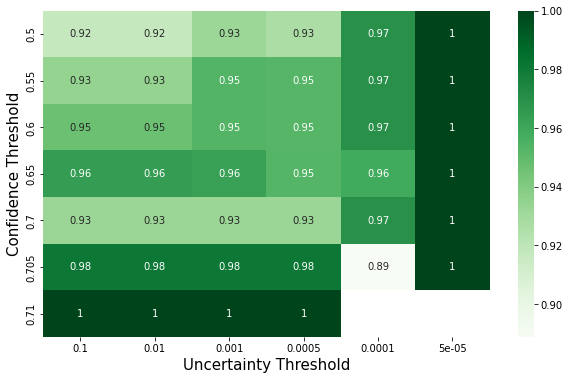


**Figure S4.** Performance evaluation by F1 score for the ProtBERT_MLP (A), ProtBERT_proto (B), ESM_MLP (C), ESM_proto (D), RoBERTa_MLP (E), and RoBERTa_proto (F) models at different confidence and uncertainty thresholds using the experimental testing dataset. F1 scores were displayed in every cell on the enzyme/plastic pairs before (top left cell in every plot) and after (other cells in every plot) filtering by the specified confidence and uncertainty thresholds.
